# S2Map: a novel computational platform for identifying secretio-types through cell secretion-signal map

**DOI:** 10.1093/bioadv/vbaf059

**Published:** 2025-03-20

**Authors:** Zongliang Yue, Lang Zhou, Peizhen Sun, Xuejia Kang, Fengyuan Huang, Pengyu Chen

**Affiliations:** Department of Health Outcomes Research and Policy, Harrison College of Pharmacy, Auburn University, Auburn, AL, 36849, United States; Department of Materials Engineering, Samuel Ginn College of Engineering, Auburn University, Auburn, AL, 36849, United States; Department of Materials Engineering, Samuel Ginn College of Engineering, Auburn University, Auburn, AL, 36849, United States; Department of Materials Engineering, Samuel Ginn College of Engineering, Auburn University, Auburn, AL, 36849, United States; Biomedical Research Department, Tuskegee University, Tuskegee, AL, 36083, United States; Department of Materials Engineering, Samuel Ginn College of Engineering, Auburn University, Auburn, AL, 36849, United States

## Abstract

**Motivation:**

Cell communication is predominantly governed by secreted proteins, whose diverse secretion patterns often signify underlying physiological irregularities. Understanding these secreted signals at an individual cell level is crucial for gaining insights into regulatory mechanisms involving various molecular agents. To elucidate the array of cell secretion signals, which encompass different types of biomolecular secretion cues from individual immune cells, we introduce the secretion-signal map (S2Map).

**Results:**

S2Map is an online interactive analytical platform designed to explore and interpret distinct cell secretion-signal patterns visually. It incorporates two innovative qualitative metrics, the signal inequality index and the signal coverage index, which are exquisitely sensitive in measuring dissymmetry and diffusion of signals in temporal data. S2Map’s innovation lies in its depiction of signals through time-series analysis with multi-layer visualization. We tested the SII and SCI performance in distinguishing the simulated signal diffusion models. S2Map hosts a repository for the single-cell’s secretion-signal data for exploring cell secretio-types, a new cell phenotyping based on the cell secretion signal pattern. We anticipate that S2Map will be a powerful tool to delve into the complexities of physiological systems, providing insights into the regulation of protein production, such as cytokines at the remarkable resolution of single cells.

**Availability and implementation:**

The S2Map server is publicly accessible via https://au-s2map.streamlit.app/.

## 1 Introduction

Cell communication is an intricate process that entails the exchange of chemicals or molecules among different cells within a specific biological environment, known as intercellular signaling (Jin *et al.* 2021, Vlahos *et al.* 2022). This process allows cells to communicate with one another, leading to a variety of outcomes, such as signal-receptor binding to recruit cells (Filippi 2019, Pajarinen *et al.* 2019), the activation of signaling pathways (Glück *et al.* 2017, Subauste 2019), and subsequent alterations in the biological functions in response to signaling events (Li 2018, Bondos *et al.* 2021, Diaz-Castro *et al.* 2021). Cell communication is crucial for pathogen recognition and the regulation of immune and inflammatory responses in the immune system. The regulation of immune and inflammatory responses is primarily through the activation of cytokines, which are the key chemical mediators in these processes (Altan-Bonnet and Mukherjee 2019, Liu *et al.* 2021). The quantification and computational characterization of cytokines become increasingly significant in both clinical medicine and biology, as these measurements facilitate the diagnosis and treatment of various diseases (Li *et al.* 2013, Nourizad *et al.* 2023). Understanding the complex regulatory mechanisms that involve diverse molecular agents requires a reductionist approach, where cells are isolated and cultivated *in vitro* (Louis *et al.* 2022). This method allows for targeted testing of specific signals, enabling researchers to measure the cellular responses accurately.

Over the past decades, substantial progress has been made in refining signal detection at the nano level (Wang *et al.* 2014, Kuang *et al.* 2017). This advancement has made it possible to measure and quantify signals emanating from individual cells, such as cytokines, within a matrix. Such high-resolution analysis not only enhances our understanding of cell-to-cell communication but also opens new avenues for therapeutic interventions. For instance, single-cell analysis techniques are now being employed to study the heterogeneity of cellular responses within a cell population, providing insights into how different cells might react to the same stimulus under varying conditions. Moreover, advancements in computational biology can be employed for modeling and simulating these complex communication networks, allowing for predicting cellular behavior in different environments and identifying potential therapeutic targets.

The characterization of spatiotemporal signals and the potential signal clustering have been inadequately addressed in current research. In a graph-based model, particularly a graph with vertices and edges, the dissymmetry measurements, such as Purchase metric (Purchase 2002) and Klaupakh metric (Klapaukh 2014), conduct a strategy to promote edge crossings to nodes with the assumption that the graph with symmetry can have a high scored axis between every pair of graph vertices (Welch and Kobourov 2017). Klaupakh metric is an advanced version of Purchase metric by overcoming its limitation of the focus on vertices, ignoring the edges that perceive as reflected across an axis even though their endpoints do not line up due to small differences in their orientation and length (Welch and Kobourov 2017). However, these metrics were still limited to network structural signal possessing and failed to perform in the complex pattern measurement at the single-cell level. The Gini coefficient (Gini 1936), initially introduced by Corrado Gini as a measure of income or wealth inequality, can also be applied to assess the relative inequality of signals, disregarding their absolute values. This provides a potential method for evaluating signal types based on dissymmetry rather than absolute signal levels. In practice, the Gini coefficient has been applied to objective functions to select optimal parameters in the domain of machine learning and signal processing (Goswami *et al.* 2018, Hehn *et al.* 2019, Hou *et al.* 2022). However, the dissymmetry of the two-dimensional (2D) signal remains challenging due to immature evaluation metrics based on the dissymmetry and diffusion.

In this paper, we present an online interactive analytical platform called cell secretion-signal map (S2Map) for intuitive exploration and interpretation of distinct cell secretion-signal patterns. To facilitate signal analysis, we introduced two new metrics, signal inequality index (SII) and signal coverage index (SCI), for a comprehensive quantitative measurement of the signal pattern in 2D space. We addressed the integration of dissymmetry-based measurement SII and diffusion-based measurement SCI for enhanced performance in distinguishing signals and cell secretion-signal patterns, which we named secretio-types. We unveil the potential cell secretio-types in the four simulation models and test them in a real-world case study of the cytokines secreted by Jurkat T cells in the immune system. Within the S2Map platform, we introduce stacked multi-layer visualization, including contour lines representing cumulative/signal changes and signal velocity, providing insights into signal flow potential. We believe that S2Map will serve as a powerful tool for investigating the complexities of physiological systems, shedding light on diverse protein production regulations in future studies.

## 2 Methods

### 2.1 SII in a two-dimensional space

The signal inequality index (SII) is designed to effectively capture the dissymmetry present in a graph’s signal distribution. A higher SII value indicates a greater degree of asymmetry in the signal. In a two-dimensional space, the SII is calculated using the Euclidean distance between the degrees of inequality (DI) derived from the signal projections on the x-axis and y-axis. This degree of inequality is determined through the Lorenz curve, which evaluates the distribution of a variable, such as the cell secretion signal in this study. A baseline representing perfect equality is established along the 45° line in the Lorenz curve, and the relative differences between the Lorenz curve and this baseline are measured.

To calculate the degree of inequality for the signal projection on the x-axis, denoted as $DI_{x}$, we applied **(1)**:


(1) $$DI_{x}=\sum_{k=1}^{n}\frac{2*\left(\frac{\sum_{1}^{k}x_{k}}{\sum_{i=1}^{n}x_{i}}-\frac{k}{n}\right)}{n},$$

where i denoted the index of pixels along the x-axis, ranging from 1 to n. The $x_{i}$ represented the values of the signal projection on the x-axis corresponding to index i. The variable k served as an index representing each position on the x-axis, and $\sum_{1}^{k}x_{k}$ symbolized the sum of signal projection values from pixel 1 to k. The $DI_{x}$ ranged from −1 to 1.

In a two-dimensional space, the SII was calculated using **(2)**, which determined the Euclidian distance between degrees of inequality calculated from the x-axis and y-axis. The SII ranged from 0 to 1, with 1 representing extreme inequality.


(2) $$\mathrm{SII}\;=\sqrt{DI_{x}^{2}+DI_{y}^{2}}.$$

### 2.2 SCI in a two-dimensional space

The signal-coverage index (SCI) is formulated as a position-specific signal-to-noise ratio (SNR) to assess the diffusion of asymmetry signals. An elevated SCI reflects a high concentration of asymmetrical signals near the central point. The SCI is determined by assessing the degree of inequality, which helps accurately pinpoint the location of these asymmetry signals.

**First**, we computed the derivative of the degree of inequality on the x-axis using the result from **(1)**. Since the pixel signal is discrete with a distance unit of 1 between neighboring pixels, we calculated the difference between the degree of inequality of adjacent pixels using **(3)**:


(3) $$\partial DI_{x(k)}=DI_{x(k)}-DI_{x\left(k-1\right)}=\frac{\sum_{1}^{k}x_{k}}{\sum_{i=1}^{n}x_{i}}-\frac{k}{n}-\left(\frac{\sum_{1}^{k-1}x_{k-1}}{\sum_{i=1}^{n}x_{i}}-\frac{k-1}{n}\right)=\frac{x_{k}}{\sum_{i=1}^{n}x_{i}}-\frac{1}{n},$$

where x(i) represented the signal value of pixel i in the x-axis, and $DI_{x0}=0.$

**Second**, we screened the count of signal units ($C_{\mathrm{signal}}$) with the $\partial DI_{x}$>0 and the count of noise units ($C_{\mathrm{noise}}$) with the $\partial DI_{x}$<=0 to derive the SNR using **(4)**. This SNR was designed to differentiate signal patterns from irrelevant signals treated as noise. This step was crucial for accurately evaluating the signals and preventing the overweighting of universally noisy or false signals detected by the biosensors.


(4) $$SNR_{x}=\frac{C_{s\mathrm{ignal}}}{C_{\mathrm{noise}}}.$$

**Third**, we generated a binary matrix by identifying positive $\partial DI_{x}$ values and assigning them signum values, while converting the negative values to 0. The signal vector ($V_{x}$) along the x-axis was then derived by multiplying the $\partial DI_{x}$ vector multiplied with the binary matrix in **(5)**. This step ensured that only the positive DI derivatives were considered, while negative DI derivatives were filtered out. This transformation effectively identified the relatively important signals and subsequently weighted them based on their positions in the later step.


(5) Vx=∂DIx(1)∂DIx(2)…∂DIx(n)*sgn∂DIx1+12sgn∂DIx1+12…sgn∂DIx1+12,

**Fourth**, we computed the center-weighted signal by the multiplication of the signal vector ($V_{x}$) with the center-weighted matrix ($W_{x}$). The center-weighted matrix was generated by assigning a weight of 1 to the center point and weights of 0 to the two endpoints. The weight distribution for the interval points along the axis followed a semicircular shape, determined using the Pythagorean theorem as outlined in **(6)**. This weighting was designed to effectively distinguish signals from different positions relative to the central cell within the matrix while ensuring robustness against signal rotation. The method of calculating the weight allowed for a consistent summation using Euclidean distance, irrespective of any rotation applied.


(6) $$W_{x(i)}=\sqrt{1-{\left(\frac{i}{n}\right)}^{2}}.$$

The axis’s center position was designated as 1 when the total pixel count was odd. In the case of an even total pixel count, two central positions were calculated using $\sqrt{1-{\left(\frac{1}{n}\right)}^{2}}$.

**Fifth**, the center-weighted signal-to-noise ratio of the x-axis, denoted as $\mathrm{CWSN}R_{x}$, were calculated by multiplying $SNR_{x}$, $V_{x},$ and $W_{x}$ in **(7)**:


(7) $$\mathrm{CWSN}R_{x}=SNR_{x}*\sum_{1}^{n}\left(V_{x(i)}*W_{x(i)}\right),$$

where i is the index of the pixels in the x-axis from 1 to n.

**Finally**, similar to the SII calculation in the two-dimensional space, the SCI were generated by the Euclidian distance between CWSNR calculated from x-axis and y-axis using **(8)**:


(8) $$\mathrm{SCI}=\sqrt{C\mathrm{WSN}R_{x}^{2}+\mathrm{CWSN}R_{y}^{2}}.$$

### 2.3 Alternative indexes for inequality measurement

We provided five alternative indexes for inequality measurement and performed a detailed comparison. These include Shannon Entropy, Theil Index, Herfindahl–Hirschman Index (HHI), Simpson’s Diversity Index, and the Atkinson Index (Table 1). We outlined their formulas and methodologies as follows.

**Table 1.** Summary of the differences across the six inequality metrics.

Metric	Range	Measures
**Degree of inequality**	[−1, 1]	Inequality with position information
**Shannon Entropy**	[0, log(n)]	Information gain
**Theil Index**	[0, ∞)	Inequality
**Herfindahl–Hirschman index**	[0, 1]	Concentration
**Simpson’s Index**	[0, 1]	Diversity
**Atkinson Index**	[0, 1]	Inequality (aversion factor)

Shannon Entropy (Shannon 1948) is effective for analyzing diversity or randomness in signal distribution based on **(9)**:


(9) $$H=-\sum_{i}p_{i}\log_{2}\left(p_{i}\right),$$

where $p_{i}$ is the proportion of the signal in each projected pixel.

Theil Index (Cowell 2000) is special for inequality in a signal distribution, decomposable into subgroups based on **(10)**:


(10) $$T=\frac{1}{n}\sum_{i}\left(\frac{x_{i}+\varepsilon }{\mu }l\mathrm{og}(\frac{x_{i}+\varepsilon }{\mu })\right),$$

where $x_{i}$ is the individual projected signal, μ is the mean, ε is a small value to prevent infinite values during log transformation, which is set to be 0.01, and n is the total number of the signals.

Herfindahl–Hirschman Index (HHI) (Kelly 1981) is useful for understanding the concentration or diversity of the signal distribution based on **(11)**:


(11) $$\mathrm{HHI}=\sum_{i}{\left(p_{i}\right)}^{2},$$

where $p_{i}$ is the proportion of the signal in each projected pixel.

Simpson’s Diversity Index (Simpson 1949) is frequently used for the diversity studies, such as the randomly selected elements are from different categories based on **(12)**:


(12) $$D=1-\sum_{i}{\left(p_{i}\right)}^{2},$$

where $p_{i}$ is the proportion of the signal in each projected pixel.

Atkinson Index (Atkinson 1970) is for inequality measurement with sensitivity adjustable via the inequality aversion parameter based on **(13)**:


(13) $$A=1-{\left(\frac{1}{n}\sum_{i}{\left(\frac{x_{i}}{\mu }\right)}^{1-\epsilon }\right)}^{\frac{1}{1-\epsilon }},$$

where ϵ is the inequality aversion parameter that is set to a default value of 0.5.

### 2.4 Simulation of the signal models in 2D space

We created a grid matrix measuring 50 by 50, initializing all positions with a signal intensity of 0. We simulated four 2D signal models: model a—Circular Propagation, model b—Circular Propagation with Direction, model c—Circular Sector, and model d—Translation.

In the model a—Circular Propagation, we initiated a signal with a height of 5000 at the central position (25,25) of the matrix. In the subsequent time point, the signal moved 4 grid units to a peripheral position, spanning grids 4 (the inner signal points) to 5 (the outer signal points) away from the matrix center. The signal decayed to 1000, which is calculated as 5000 divided by the distance from the center (a distance equal to 5 in this example). This shifting process was repeated for the third and fourth time points.

For model b—Circular Propagation with Direction, we followed the same steps as in model a—Circular Propagation, but with the added consideration of signal direction. The signal shifted to the right by four grid units at each time point.

For the model c—Circular Sector, we generated a grid matrix with x-axis and y-axis coordinates ranging from 0 to 50. To create a distance matrix, we subtracted the center point coordinates (25,25) from the grid matrix, resulting in x-axis and y-axis distances ($d_{x}$ and $d_{y}$) that varied from −25 to 25. Subsequently, we implemented coordinate filtering based on the conditions $d_{x}\geq d_{y}$ and $d_{x}\geq -(d_{y}+1)$. This filtering masked all coordinates except those within a sector pointing to the right.

For model d—Translation, we implemented a process where the signal is translated from the center to the right by four grid units at each time point. The signal decay followed a pattern similar to the three aforementioned models.

We performed rotations from 0° to 330° in 30° increments to test the robustness of the one-dimensional metrics (DI and CWSNR) and two-dimensional metrics (SII and SCI) against rotations in the model d—Translation.

### 2.5 Robustness of SII and SCI to the signal pattern scale and rotation

The readouts of graph signals in a 2D matrix were scaled depending on the resolution of the detection probes. In the design of the SII and SCI, the scale effect was regulated based on relative measurement (Saaty 1993). In the SII calculation, the difference between cumulative normalized signals and the baseline of perfect equality was normalized by dividing the total number of pixels. Consequently, when the signal height and relative position remained unchanged, the SII remained constant. For example, both DI for the signals of [0,0,0,0,0,0,0,1,0,0] and [0,0,0,0,0,0,0,0,0,0,0,0,0,0,1,1,0,0,0,0] were −0.5. Therefore, SII remained the same as derived from DI. Similarly, in the SCI calculation accounting for the scaling effect, when the size of the signal vector ($V_{x}$) increased, the sum of the signal vector ($V_{x}$) remained constant, and the SNR remained the same. Although the weights varied due to the estimated mean of weight on a small scale $\sqrt{1-{\left(\frac{i}{n}\right)}^{2}}$ being smaller than the weight for a double size $\frac{1}{2}\sqrt{1-{\left(\frac{2i-1}{2n}\right)}^{2}}$+$\frac{1}{2}\sqrt{1-{\left(\frac{2i}{2n}\right)}^{2}}$, the trend of differences tended to be ignored when n is extreme large. Therefore, the SCI remained the same regardless of the changes in signal map resolution. Additionally, the SII and SCI remained constant even when the signal pattern rotated, as the calculation relied on Euclidean distance. The robustness to signal rotation is critically important in identifying the different secretio-types.

### 2.6 Synthetic data with varying signal dimensions for scalability and efficiency testing

We generated matrices with dimensions exponentially increasing by a factor of 2, starting from 400 × 400 and scaling up to 12 800 × 12 800. For each matrix size, we repeated the tests 100 times, using random seeds ranging from 0 to 99. Execution times were recorded using Python’s time module, and the memory peaks were captured using Python’s tracemalloc module. The performance was evaluated by the mean and standard deviation of execution time and memory usage. The tests were performed in an environment equipped with an Intel Core i7-7700HQ Processor.

### 2.7 The signal visualization using the stacked three-layer

The signal interpolated heatmap layer presents a 2D heatmap generated by the Radial Basis Function (RBF) with a 2D Gaussian function as default. To enhance the signal’s surface smoothness, signal interpolation was applied using the interpolate. Rbf function of the SciPy library. S2Map provided alternative functions, including a multiquadric, inverse, linear, cubic, quintic, and thin plate for signal interpolation in the 2D heatmap. Therefore, the signal delta changes, representing the increments from previous time points in the time-series data, were visualized using the signal interpolated heatmap layer.

To enhance the visualization of signal patterns, S2Map incorporated signal interpolated heatmap’s contour line layer, showing both cumulative signal and signal delta change’s contour lines. We followed the same procedure used for the signal interpolated heatmap layer to generate the signal surface. Based on the gradient in the signal surface, contour lines were created to stratify potential signal patterns at different cutoffs. These contour lines allowed users to preview the signal patterns and adjust the signal cutoff accordingly, effectively zeroing out regions considered as noise.

The signal velocity layer is based on a metric that gauges the direction of signal flow potential within asymmetrical signal patterns. S2Map granted users to select the number of hotspots, such as peaks and valleys, based on the order of all signals. Subsequently, S2Map masked the other grid to generate the gradients. In particular, we applied the same step mentioned in the signal interpolated heatmap layer to generate the signal surface. After obtaining the smoothed signals, we computed the smoothed signal gradients in a 2D array. These gradients were visualized in the stream plot by associating the peak-hotspot positions with the starting points of the arrows using the streamplot function of the matplotlib library. Consequently, the signal velocity can be depicted as arrows pointing from the signal peaks to the valleys on a smoothed signal surface.

### 2.8 The real-world case study on single-cell cytokine secretion

The fabrication of LSPR imaging (LSPRi) sensors followed a previously established procedure (Zhou *et al.* 2023) with modifications. A microfluidic flow-patterning polydimethylsiloxane (PDMS) layer with parallel microfluidic channels [400 μm (W) × 2.5 cm (L) × 50 μm (H)] was fabricated using deep reactive-ion etching. The PDMS layer was attached to a thoroughly cleaned glass slide after plasma treatment. A 100 μg/ml poly-D-lysine (PDL) solution was injected into the channel and incubated for 1 hour at room temperature. A 0.05 mg/ml citrate-capped gold nanospheres solution (Ø = 50 ± 4 nm) was then flowed into the channel for 10 minutes and incubated overnight at 4°C to allow for immobilization in the channel. A 1 μg/ml anti-IL-6 peptide aptamer solution (Scheme 1) was loaded for 10 minutes and incubated for 1 hour. The sensor surface was subsequently passivated with 1% casein in PBS for 1 hour before being applied for the LSPRi immunoassay. The LSPRi sensors were placed under a dark-field microscope with an EMCCD camera. The intensity of scattered light from the gold nanospheres was recorded in real-time. A 500 cell/ml solution of Jurkat T cells in a mixed stimulus of PMA (0.1 μg/ml) and Ionomycin (1 μg/ml) was loaded into the channel. The Jurkat T cells were pre-stained with 0.1 μmol/l Calcein AM. After the flow was stopped, the fluorescently labeled single cells were suspended above the LSPRi sensors. One of these single cells was located, and a fluorescent image was obtained while real-time LSPR images were captured every 10 seconds.

### 2.9 The S2Map web application

We utilized streamlit.io (https://streamlit.io/) in conjunction with the streamlit library (https://pypi.org/project/streamlit/) for the development of the front-end user interactive platform. The resulting streamlit application was deployed on streamlit.io’s community cloud (https://streamlit.io/cloud). Real-time computational analysis involving SII and SCI was conducted using Python. The 3D plot was created using a Python library, the matplotlib, for graph visualization. S2Map integrated the three visualization layers to generate signal maps. Additionally, all figures and tables were downloadable, featuring clickable support through the download function in the streamlit library.

## 3 Results

### 3.1 The signal measurement through DI and CWSNR in one-dimensional space

**Figure 1.**
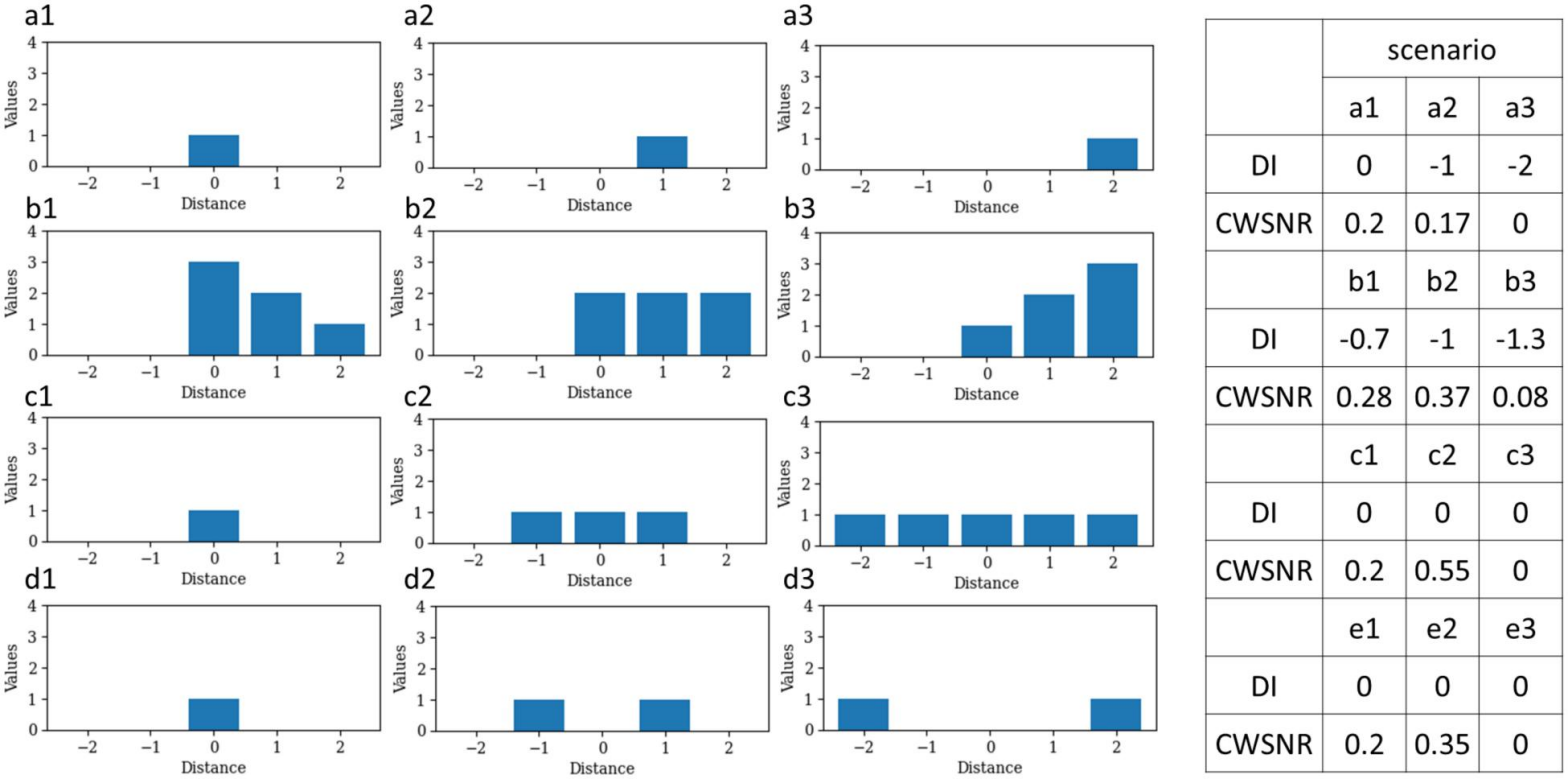
The simulated one-dimensional signals exhibiting dissymmetry quantified by DI and the signal scale quantified by CWSNR. We conducted simulations within a 5-pixel space, with the center designated as 0 (subgraph on the left side). The four simulated models, including model a—Signal Shifting: shifting the signal to the right side, model b—Signal Shaping: introducing signals with varying value distributions in the right 3 pixels, model c—Signal Spreading: dispersing the signal from the center to encompass the center 3 pixels and eventually all pixels, and model d—Signal Propagating: initiating the signal from the center and propagating it towards the periphery, reaching both end sides. We measure the DI values and CWSNR values to showcase the differences among the four simulated models.

In the one-dimensional simulated signal measurements, we observed that DI and CWSNR could complement each other to improve signal identification (Fig. 1). The asymmetric signals within the 5-pixel space in the simulated models (a—Signal Shifting and b—Signal Shaping) can be characterized and quantified using DI, ranging from −1 to 1. Meanwhile, CWSNR gauged signal scale, showcasing its capacity to recognize the central and peripheral signal positions in the model a—Signal Shifting. CWSNR could sensor the signal changes in the simulated models c—Signal Spreading and d—Signal Propagating since it is calculated by the product of weighted distance scores and the SNR. While the SNR remained constant, the weighted distance score varied from the center to the periphery, dominating the distinction.

In model b—Signal Shape, we noted that when signals were distributed as the densities of 3, 2, and 1 within a 5-pixel space, SNR in the derivative of the degree of inequality changed to 2/3. The signal density of 1 was considered noise, displaying a negative value in the derivative of the degree of inequality. Consequently, b2 exhibited the highest CWSNR value, followed by b1, with b3 ranking last.

For both the c—Signal Spreading and d—Signal Propagation models, DI consistently showed 0, while CWSNR effectively distinguished differences from both the signal scale and position perspectives. In c—Signal Spreading, CWSNR employed SNR to elevate the signal at timepoint c2 and assigned a value of 0 to the uniform distributed signal at the timepoint c3. In d—Signal Propagation, CWSNR weighs the differences between the time points d2 and d3 using the center-weighted matrix to increase the signal weights near the center point.

**Figure 2.**
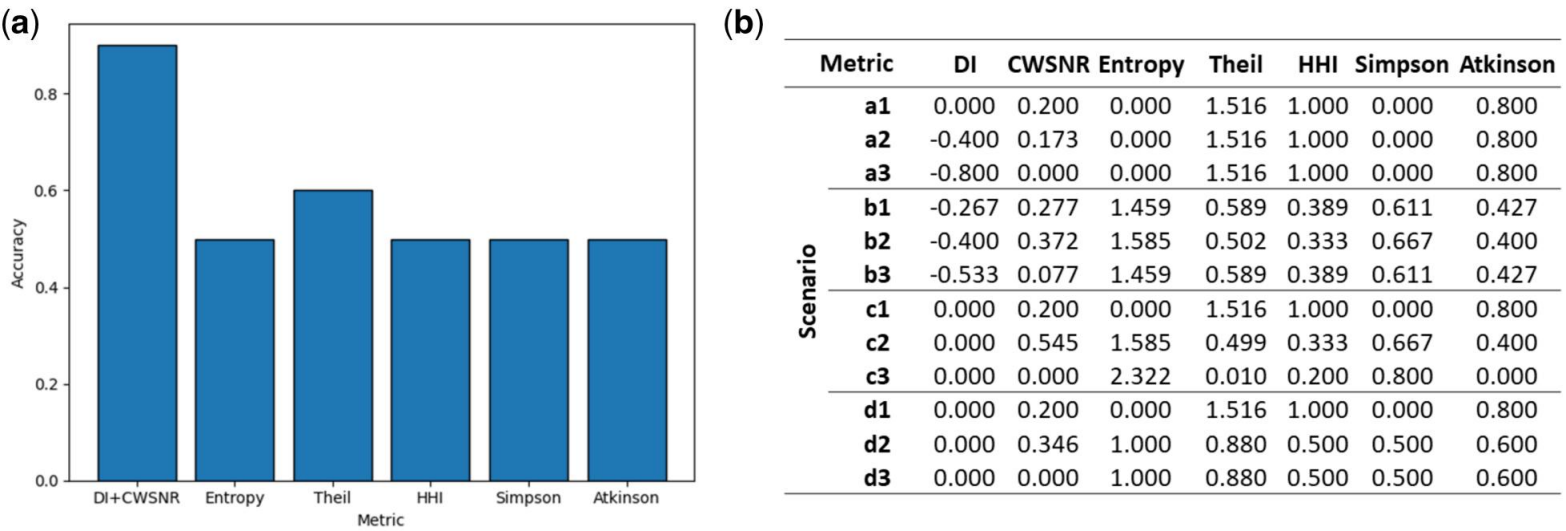
The performance of the metrics in distinguishing different scenarios in the simulated one-dimensional signals. (a) The accuracy of separating the scenarios based on metric outcomes. (b) The metric outcomes for the different scenarios in the simulated one-dimensional signal models.

In the performance comparison among the six metrics, we found that the DI and CWSNR achieved the best results in distinguishing the 10 unique scenarios in the simulated one-dimensional signals, with an accuracy of 0.9 (Fig. 2). These metrics outperformed the other five in recognizing signal position changes in the model a—Signal Shifting. Additionally, the CWSNR complemented the DI by effectively recognizing signal coverage changes. In contrast, the Shannon Entropy, Theil Index, HHI, Simpson, and Atkinson metrics failed to recognize the signal position changes.

### 3.2 The signal measurement through SII and SCI in two-dimensional space

**Figure 3.**
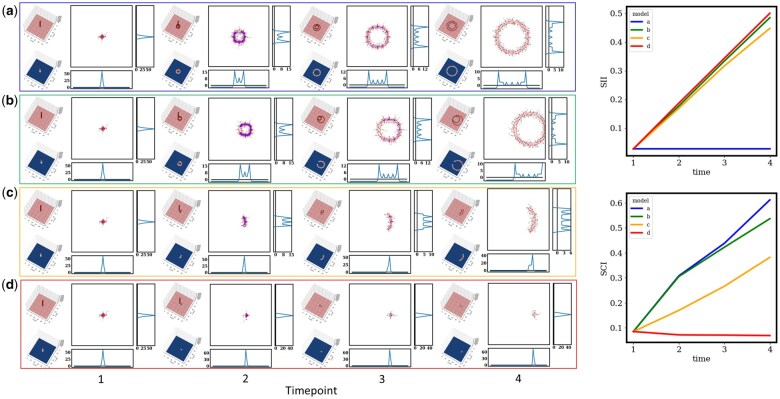
Two-dimensional simulated signals showcase signal dissymmetry, characterized by SII, and the signal’s scale is assessed using SCI. Simulations were carried out within a 51 × 51 pixel space, with the *center* defined as (25,25). The four simulated scenarios encompass: a—Circular Diffusion: signal spreading in a circular pattern, model b—Circular Diffusion with Direction: signal diffusion in mode a circular pattern shifting to the right side, model c—Circular Sector: signal diffusion within a circular section towards the right side, and model d—Translation: signal diffusion in a linear pattern extending to the right side.

In our two-dimensional signal simulation, the SCI exhibited enhanced capabilities in detecting changes within signal patterns at a finer resolution, complementing the performance of the SII (Fig. 3). In the SCI value ranking, the model d—Translation was ranked first, aligning with the findings from the one-dimensional model a—Signal Shifting, owing to its notable degree of inequality. Following closely was model b—Circular Diffusion with Direction, wherein signals from the x-axis projection skewed towards the right, featuring a prominent peak at the extreme right. The model c—Circular Sector was placed in the third position, with its signal peak shifting towards the right-middle region. Notably, the model d—Translation ranked last, scoring 0, indicating a perfect symmetry in signal patterns. Therefore, the SII could distinguish the signal inequality in the four models.

In the SCI time-series plot, the model a—Circular Diffusion emerged as the highest ranked by SCI values. This was attributed to an overall increase in signal amplitude over time and the presence of a signal peak consistently positioned around the center position of the matrix. Following closely was the model b—Circular Diffusion with Direction, exhibiting a gradual increase in signal patterns over time, with a dispersion towards the edge. The third-ranked mode was the model c—Circular Sector, characterized by a partial increase in signal area along the y-axis. Finally, the model d—Translation occupied the last position, as its signal area remained constant in size, shifting to a peripheral position with a lower weight score. Therefore, SCI could detect the position-specific signal coverage changes for the four different models.

### 3.3 The robustness testing against the rotation of two-dimensional metrics and one-dimensional metrics

The two-dimensional metrics, SII and SCI, outperformed the one-dimensional metrics in detecting signal shifts and showed minimal influence from signal rotations (Table 2). Specifically, DI failed to detect changes in signal position from the center, as the means of DI at time points 2, 3, and 4 of the model d—Translation remained at 0. Additionally, DI exhibited variance with rotations, and this variance tended to increase as the signal moved farther from the center point, indicating a strong dependence on rotation. In contrast, SII successfully detected signal translation relative to the center point and demonstrated robustness against rotation with minimal variance.

**Table 2.** The model d—Translation signal rotation impacts the one-dimensional and two-dimensional metrics.

Scenario	d2													

**Rotation**	0	30	60	90	120	150	180	210	240	270	300	330	Mean	Variance
$DI_{x}$	−0.19	−0.17	−0.11	−0	0.08	0.15	0.19	0.17	0.11	0.02	−0.08	−0.15	0.00	0.02
$\mathrm{CWSN}R_{x}$	0.04	0.06	0.06	0.06	0.06	0.06	0.04	0.06	0.06	0.06	0.06	0.06	0.06	6.95E−05
**SII**	0.19	0.19	0.19	0.19	0.19	0.19	0.19	0.19	0.19	0.19	0.19	0.19	0.19	1.13E−07
**SCI**	0.07	0.09	0.09	0.07	0.09	0.09	0.07	0.09	0.09	0.07	0.09	0.09	0.08	5.51E−05

**Scenario**	**d3**													

**Rotation**	0	30	60	90	120	150	180	210	240	270	300	330	Mean	Variance

$DI_{x}$	−0.35	−0.31	−0.19	−0	0.16	0.29	0.35	0.31	0.19	0.02	−0.16	−0.29	0.00	0.06
$\mathrm{CWSN}R_{x}$	0.04	0.06	0.06	0.06	0.06	0.06	0.04	0.06	0.06	0.06	0.06	0.06	0.06	7.6E−05
**SII**	0.35	0.35	0.35	0.35	0.35	0.35	0.35	0.35	0.35	0.35	0.35	0.35	0.35	1.6E−07
**SCI**	0.07	0.08	0.09	0.07	0.08	0.09	0.07	0.08	0.09	0.07	0.08	0.09	0.08	5.0E−05

**Scenario**	**d4**													

**Rotation**	0	30	60	90	120	150	180	210	240	270	300	330	Mean	Variance

$DI_{x}$	−0.51	−0.45	−0.27	−0	0.24	0.43	0.51	0.45	0.27	0.02	−0.24	−0.43	0.00	0.13
$\mathrm{WSN}R_{x}$	0.03	0.05	0.06	0.06	0.06	0.06	0.03	0.05	0.06	0.06	0.06	0.06	0.05	8.65E−05
**SII**	0.51	0.51	0.51	0.51	0.51	0.51	0.51	0.51	0.51	0.51	0.51	0.51	0.51	1.50E−07
**SCI**	0.07	0.08	0.08	0.07	0.08	0.08	0.07	0.08	0.08	0.07	0.08	0.08	0.08	3.69E−05

### 3.4 The scalability and efficiency testing

**Figure 4.**
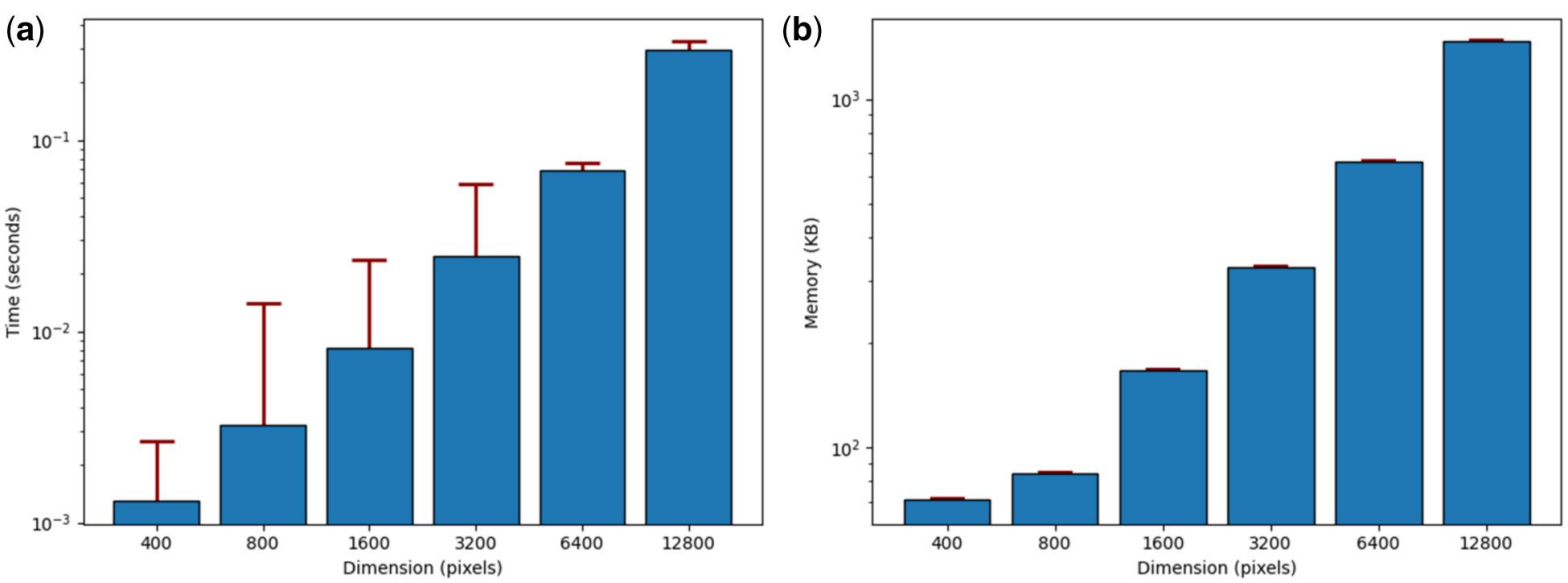
The execution time and memory usage in processing the synthetic signal matrices with varying dimensions. Tests were conducted using synthetic signal matrices with dimensions increasing exponentially by a factor of 2, starting from 400 × 400 and scaling up to 12 800 × 12 800, to assess (a) the execution time measured in seconds and (b) the peak memory usage measured in kilobytes (KB). The error bar represents standard deviation.

We conducted scalability and efficiency testing for SII and SCI calculations using synthetic signal matrices with varying dimensions (Fig. 4). The results demonstrated that execution time scales linearly with matrix dimensions. For 100 repeated tests using matrices with dimensions of 12 800 × 12 800, the average execution time was 0.3 seconds with a standard deviation of 0.03 seconds, while the peak memory usage averaged 1.4 MB with minimal variance.

### 3.5 The S2Map platform user interface

**Figure 5.**
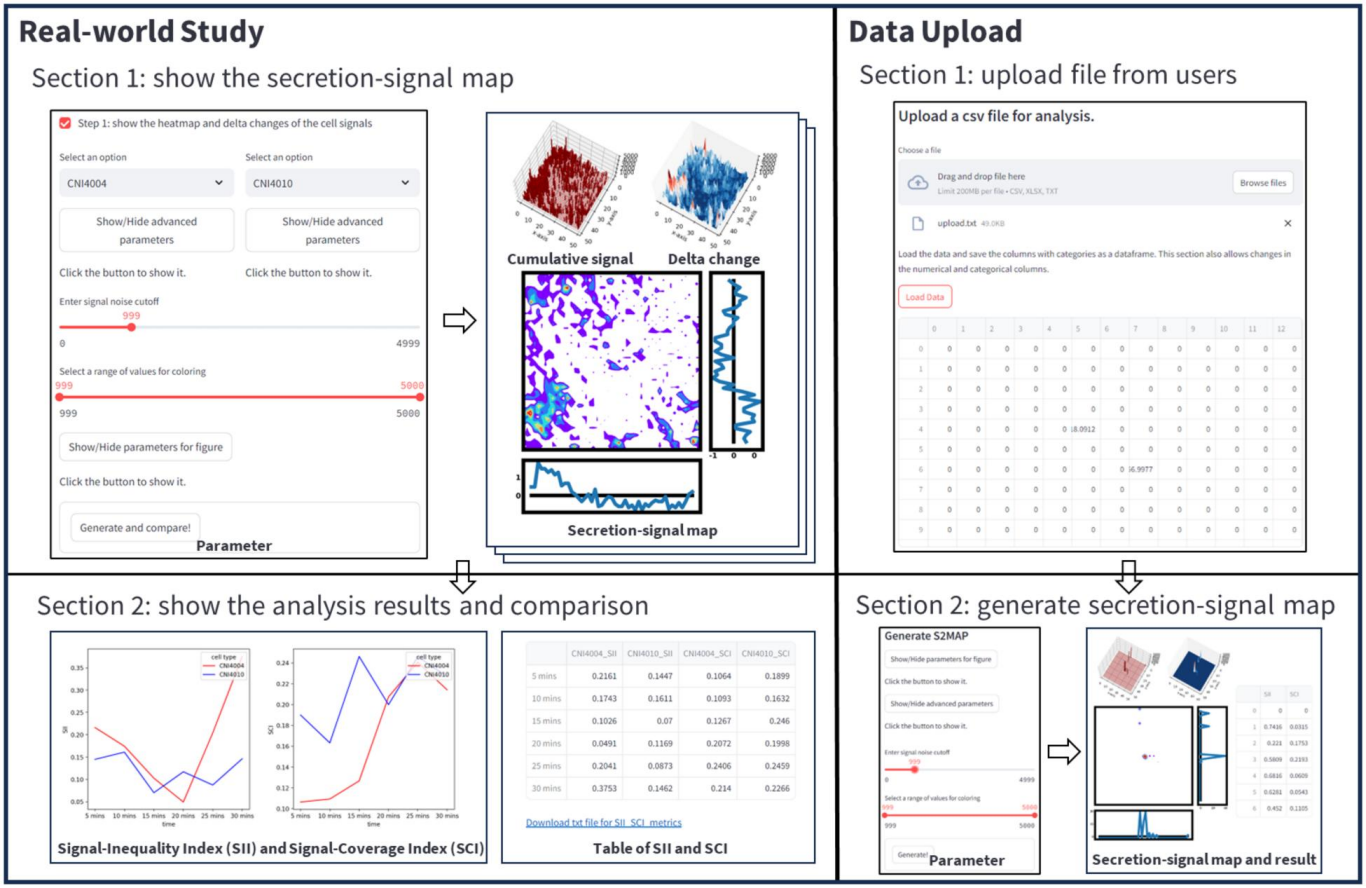
The user interface for signal secretion map generation and signal comparison is divided into two main panels. The Real-World Study panel focuses on cell secretion-map generation and signal analysis results. In section 1, there are 10 advanced parameters, including options for selecting interpolation function, specifying the number of hotspot nodes, toggling stream plot arrows, adjusting arrow density, displaying signal interpolated heatmap’s contour lines and cumulative signal contour lines, setting the cutoff for cumulative signal contours, and selecting the center coordinates and diameter. The graphics panel presents a cumulative 3D signal figure, a 3D signal delta change figure, and a 2D secretion-signal map. In section 2, the signal comparison is performed using SII and SCI metrics. S2Map generates two graphs illustrating the temporal changes in SII and SCI, and the results are provided in a table with an attached downloadable link. The Data Upload panel allows users to visualize and analyze signals using their uploaded data. Users can upload their signal matrix via the data upload page by either dragging and dropping their file or clicking the “Browse File” option. The matrix can be either n×n (n rows and n columns) or a m×n (m=x×n with x time points in a time series). After clicking the “Load Data” button, the matrix will be displayed in the table below. Users can adjust parameters as needed. By clicking the “Generate” button, a secretion signal map will be produced, along with a downloadable table containing the SII and SCI results.

The S2Map platform consists of two major panels (Fig. 5). The first panel is the real-world study of the immune cell secretion-signal dataset. The second panel is the user data upload.

The first panel has two sections: the visualization of the secretion-signal map and the signal analysis results for comparison. The first section, S2Map visualization, provides a parameter setting and graph panel. The parameter setting panel allows users to adjust two basic parameters: the signal noise cutoff and the value range for coloring. Additionally, 10 advanced parameters are available for refining the visualization of the secretion-signal map, along with six parameters dedicated to figure customization, such as resolution and label size. The graph panel will display the results by clicking the “Generate and compare!” button. S2Map generates three subfigures for each time point. The two smaller 3D figures at the top illustrate cumulative signals and signal delta changes. The signal delta changes are based on the current timepoint signals minus the previous timepoint’s signals. In particular, the cumulative 3D signal figure uses two color bars: grey represents the baseline signal from the previous timepoint, while red on top indicates the delta change. The bottom figure shows the 2D secretion-signal map with inequalities based on signal projections along the x and y axes. In the second section, signal analysis results for comparison, S2Map will generate the SII and SCI results and plot the temporal changes, allowing for a comparison between the two selected samples.

In the second panel, S2Map provides a data upload option for users to generate their own secretion-signal map. Users can upload a matrix in a plain text file, whether it is a single timepoint matrix or a time-series matrix. Like the first panel, S2Map will generate the signal maps with the SII and SCI results.

### 3.6 The real-world case study of IL-6 secretion of Jurkat T cells

In the real-world case study of interleukin 6 (IL-6) secretion, we collected IL-6 signals via peptide binding over a 30-minute period. To account for signal fluctuations, we averaged the signals within a 1-minute window preceding each time point, including the selected time point itself. The signal matrix at the 0 minute was used as the baseline to neutralize noisy signals. If a specific time point had a lower signal than at the 0 minute, that time point was used as the baseline instead. The signal delta changes were then used to generate SII and SCI values.

**Figure 6.**
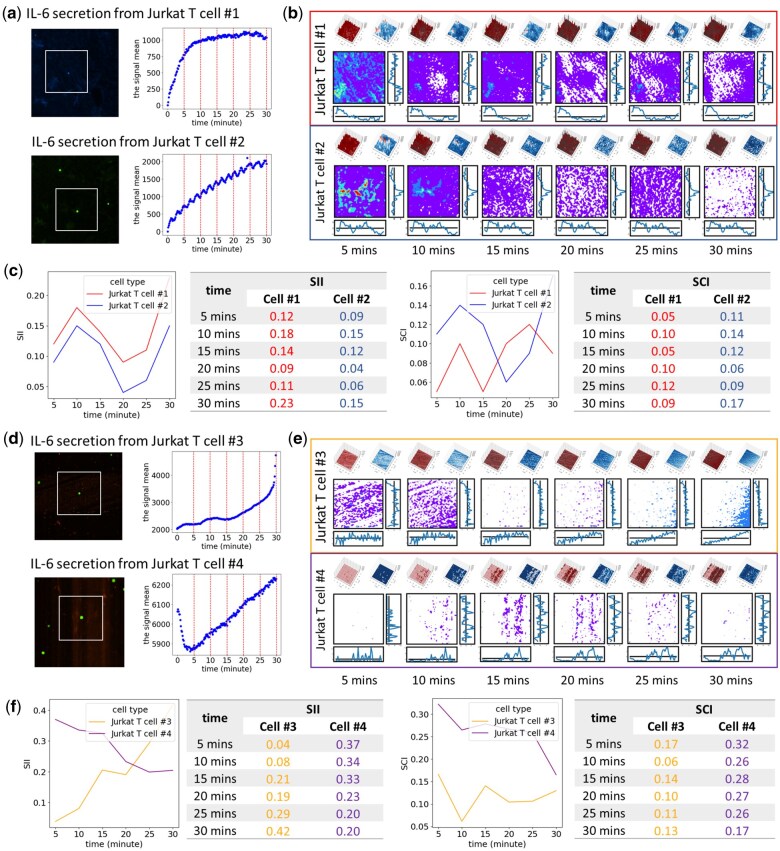
An example of the IL-6 secretion signals from two pairs of Jurkat T cells presenting signal temporary changes. (a, d) Identification of IL-6 secretion signals (red fluorescence) from two independent single cells and their mean signal intensity over time. The full field of view consists of 521 × 521 pixels, with a highlighted region of 250 × 250 pixels containing a captured Jurkat T cell (green fluorescence) at the center. The final signal matrix is aggregated into a 50 × 50 grid using 5 × 5 blocks for S2Map analysis. (b, e) Visualization of the signal pattern using S2Map. (c, f) Temporal changes in the signal inequality index (SII) and signal coverage index (SCI).

S2Map analysis indicated that the temporal SII values of Jurkat T cell #1 and Jurkat T cell #2 were highly similar (Fig. 6). In this comparison, the signal noise cutoff was set to 0. The Pearson correlation coefficient of the temporal SII values of Jurkat T cell #1 and Jurkat T cell #2 was 0.94 with a *P*-value of 0.006. In contrast, the SCI analysis revealed that while the signal coverage of both cells remained relatively stable during the first 15 minutes, it diverged significantly between 15 and 30 minutes. During this period, the SCI value of Jurkat T cell #1 decreased, indicating that its signal became more concentrated in the central region, whereas the SCI value of Jurkat T cell #2 increased, suggesting that its signal became more dispersed toward the peripheral areas. Furthermore, the strong correlation between SII and SCI trends in Jurkat T cell #2, with a Pearson correlation coefficient of 0.95 and a *P*-value of 0.004, suggested that signal inequality played a more dominant role in shaping secretion patterns than signal distribution, which exhibited a relatively small variance of SNR values and no significant signal shifting.

S2Map analysis indicated that the temporal SII values of Jurkat T cell #3 and Jurkat T cell #4 were highly dissimilar (Fig. 6). In this comparison, the signal noise cutoff was set at 10% of the peak signal for delta changes, corresponding to 444 for Jurkat T cell #3 and 389 for Jurkat T cell #4. The Pearson correlation coefficient between their SII values was −0.86 with a *P*-value of 0.028, indicating a strong negative correlation. Jurkat T cell #3 initially exhibited a symmetric signal pattern with a low SII value, but the delta signals became increasingly concentrated in the right bottom corner, resulting in a high SII value over time. Conversely, Jurkat T cell #4 started with an asymmetric signal pattern, with peak signals concentrated at the right bottom position. However, the signal distribution shifted toward a more symmetric pattern, with signals becoming evenly distributed over time.

**Figure 7.**
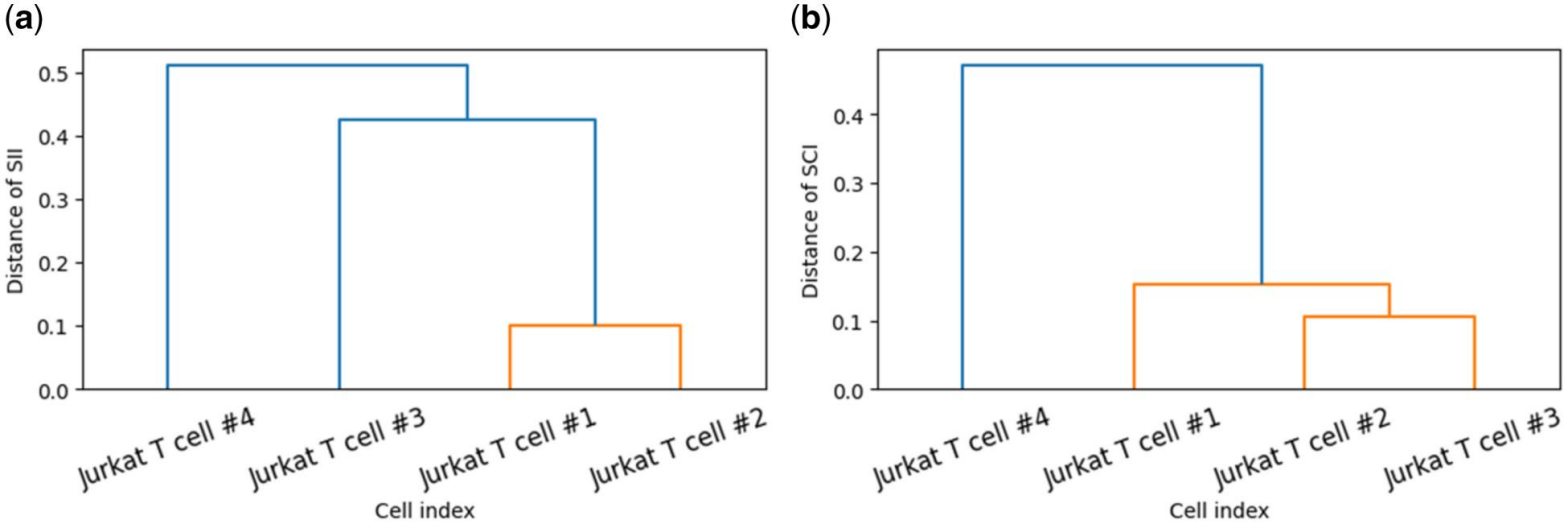
Hierarchical clustering of four Jurkat T cells based on (a) SII and (b) SCI values. The distances for SII and SCI are calculated using the Euclidean distance of these values from temporal signal changes.

S2Map analysis examined potential spatial differences in secretion signals between two groups of Jurkat T cells: cells #1, #2, and #3, and cell #4. Hierarchical clustering based on SII and SCI values (Fig. 7) revealed that Jurkat T cell #4 exhibited lower similarity to the other three cells, driven by consistently higher SII and SCI values over time. This suggests a more anisotropic secretion pattern in Jurkat T cell #4 compared to the isotropic secretion observed in Jurkat T cells #1, #2, and #3.

Overall, the Jurkat T cell IL-6 secretion study demonstrated the utility of S2Map in quantifying dynamic signal patterns and enabling comparative analysis of cell secretion trends over time. Notably, cells could exhibit highly similar secretion correlations over time, even if their individual signal patterns were not identical, as observed in the comparison between Jurkat T cell #1 and Jurkat T cell #2. Conversely, secretion patterns might follow distinctly different temporal trends, even among cells of the same type, as seen in the comparison between Jurkat T cell #3 and Jurkat T cell #4. Combining SII and SCI values could provide insights into the secretio-types of cells based on both temporal and spatial secretion patterns.

## 4 Discussion

The development of cell signal-secretion analysis tools highlights the critical need for integrating experimental and computational approaches to decode the complex language of cell communication. This study introduces S2Map, a novel platform for exploring and interpreting cell secretion-signal patterns through quantitative assessments of dissymmetry and diffusion. S2Map effectively bridges the gap between traditional *in vitro* single-cell secretion experiments and advanced quantitative analyses, facilitating the identification of distinct secretion patterns. By enabling precise measurement and characterization of secretion-type signals, S2Map provides researchers with a powerful tool to unravel the intricate patterns of cell secretion. This deeper understanding is crucial for decoding how cells interact within their microenvironment, especially in contexts where cell communication is altered or disrupted. The insights gained from using S2Map can significantly enhance the development of more effective diagnostic tools and therapeutic strategies for diseases characterized by aberrant cell communication, such as cancer, autoimmune disorders, and infectious diseases.

The two indices introduced in S2Map, SII and SCI, provide a powerful means to detect differences in our simulation models. While the current study focuses on 2D signal inequality measurements, future work will extend these capabilities to 3D and multi-dimensional analyses, particularly in capturing inequalities within stereo secretion signal detection. In a real-world case study examining IL-6 cytokine secretion from Jurkat T cells, we demonstrate the SII and SCI utility in discovering the secretion signal dynamics. S2Map enhances the visualization of these signals by employing both 2D and 3D plots. Notably, the secretion-signal map features advanced multi-layered visualization techniques, including a signal-interpolated heatmap layer, a signal-interpolated heatmap’s contour line layer, and a signal velocity layer. These layers work in tandem to improve the visual detection and analysis of complex signal patterns, enabling a more comprehensive understanding of cellular behavior.

The Jurkat T cell IL-6 secretion study highlights S2Map’s ability to quantify dynamic signal patterns and enable comparative analysis of secretion trends over time, potentially revealing heterogeneity in secretion signals within the extracellular space. IL-6 is a critical yet controversial cytokine within the inflammatory and cancer microenvironments, underscoring the importance of understanding the direction of its secretion for influencing subsequent cytokine secretion or activity of surrounding cells (Hirano 2021). For example, in leukemia and solid tumors, where numerous macrophages and monocytes are present, Jurkat T cells can interact with macrophages through ligand–receptor interactions (Chanput *et al.* 2015, Cassetta and Pollard 2018, Wang and Zheng 2019). When IL-6 secreted by Jurkat T cells is uniformly distributed, it may evenly influence surrounding macrophages and further stimulate or enhance the release of pro-inflammatory cytokines by monocytes. When surrounding monocytes are activated, they can educate the surrounding stromal cells to form pro-tumor cells, highlighting the importance of determining the secretion direction. However, if the cells are anisotropically distributed, IL-6 will primarily affect the macrophages near the cytokine secretion site. Consequently, selecting therapeutic compounds that target activated monocytes could achieve more precise treatment.

Furthermore, S2Map is capable of integrating user’s upload data with advanced computational models and extends its application to a wide range of similar challenges, such as the gene transcriptomic patterns in single-cell latent space (Pezzotti *et al.* 2017, McInnes and Healy 2018, Do and Canzar 2021, Giraldo *et al.* 2021, Ujas *et al.* 2023) or spatial single-cell transcriptomic analysis (Nagasawa *et al.* 2021, Ho *et al.* 2022, Park *et al.* 2022, Maier *et al.* 2023). S2Map can be extended to quantify spatial heterogeneity across different biological conditions, particularly in spatial single-cell transcriptomic analysis. This allows for the prioritization of underlying genes that exhibit distinct patterns in single-cell spatial profiles. This integration not only accelerates the discovery of novel signal patterns but also enables customized signal analysis across various types of 2D signals. As we continue to refine these tools and extend the potential for identifying secretio-types, S2Map will offer new possibilities for targeted treatments that address the root causes of diseases linked to dysfunctional cell communication. We envision S2Map becoming an essential tool for investigating the complexities of physiological systems, providing valuable insights into diverse signal regulations in future research.

## 5 Conclusions

The S2Map represents a significant advancement in computational cell secretion-signal analysis, bridging the gap between traditional experimental approaches and advanced quantitative assessments. By introducing the SII and SCI, S2Map provides a powerful framework for identifying and interpreting distinct secretion patterns at the single-cell level. The platform’s multi-layer visualization capabilities enhance the detection and characterization of secretion dynamics, offering valuable insights into cellular communication mechanisms. In a real-world case study of IL-6 secretion from Jurkat T cells, S2Map effectively distinguished temporal and spatial secretion trends, demonstrating its potential in biomedical research. Moving forward, S2Map's adaptability to 3D and multi-dimensional analyses will further expand its applicability, fostering deeper exploration of cellular behaviors and their implications in disease physiology.

## Author contributions

Conceptualization: Zongliang Yue and Pengyu Chen; Methodology: Zongliang Yue, Lang Zhou, and Fengyuan Huang; Formal analysis: Zongliang Yue and Lang Zhou; Investigation: Zongliang Yue, Lang Zhou, Peizhen Sun, and Xuejia Kang; Writing—original draft preparation: Zongliang Yue, Lang Zhou, and Xuejia Kang; Writing—review and editing: Zongliang Yue, Lang Zhou, Xuejia Kang, Fengyuan Huang, and Pengyu Chen; Visualization: Zongliang Yue; Supervision, Zongliang Yue and Pengyu Chen; Project administration: Zongliang Yue and Pengyu Chen; Funding acquisition: Zongliang Yue and Pengyu Chen. All authors have read and agreed to the published version of the manuscript.

## Conflict of interest

The authors declare that the research was conducted in the absence of any commercial or financial relationships that could be construed as a potential conflict of interest.

## Funding

This work has been supported by the funding from the National Science Foundation (NSF) CAREER (grant number CBET-1943302 to P.C.) and National Institutes of Health (NIH) MIRA (grant number 2R35GM133795 to P.C.).

## Data Availability

Supplementary data are available on the S2Map server online.
